# Developmental Stage- and Genotype-Dependent Regulation of Specialized Metabolite Accumulation in Fruit Tissues of Different Citrus Varieties

**DOI:** 10.3390/ijms20051245

**Published:** 2019-03-12

**Authors:** Roya Nadi, Behrouz Golein, Aurelio Gómez-Cadenas, Vicent Arbona

**Affiliations:** 1 Faculty of Agriculture, Tabriz University of Tabriz, Tabriz 5166616471, Iran; r.nadi1366@gmail.com; 2 Citrus and Subtropical Fruits Research Center, Ramsar 4691733113, Iran; b.golein@areo.ir; 3 Department de Ciències Agràries i del Medi Natural, Universitat Jaume I, 12071 Castelló de la Plana, Spain; aurelio.gomez@camn.uji.es

**Keywords:** bitterness, citrus genotypes, flavonoids, harvesting time, limonoids, metabolomics

## Abstract

Flavor traits in citrus are the result of a blend of low molecular weight metabolites including sugars, acids, flavonoids and limonoids, these latter being mainly responsible for the characteristic bitter flavor in citrus. In this work, the genotype- and developmental stage-dependent accumulation of flavonoids and limonoids is addressed. To fulfill this goal, three models for citrus bitterness: bitter Duncan grapefruit, bittersweet Thomson orange and sweet Wase mandarin were selected from a total of eight different varieties. Compounds were annotated from LC/ESI-QqTOF-MS non-targeted metabolite profiles from albedo and pulp tissues. Results indicated that the specific blend of compounds providing the characteristic flavor trait is genotype-specific and hence under genetic control, but it is also regulated at the developmental level. Metabolite profiles in albedo mirrored those found in pulp, the edible part of the fruit, despite differences in the concentration and accumulation/depletion rates being found. This is particularly relevant for polymethoxylated flavones and glycosylated limonoids that showed a clear partitioning towards albedo and pulp tissues, respectively. Fruit ripening was characterized by a reduction in flavonoids and the accumulation of limonoid glycosides. However, bitter grapefruit showed higher levels of limonin A-ring lactone and naringin in contrast to sweeter orange and mandarin. Data indicated that the accumulation profile was compound class-specific and conserved among the studied varieties despite differing in the respective accumulation and/or depletion rate, leading to different specialized metabolite concentration at the full ripe stage, consistent with the flavor trait output.

## 1. Introduction

The characteristic citrus flavor and taste is predominantly governed by the concentration of soluble solids including metabolizable and structural carbohydrates and titrable acids (namely citric acid but also minor amounts of other organic acids) [1]. Citrus are also widely acknowledged as rich sources of a vast array of minor metabolites with specialized functions within tissues and cells such as pest and microbial defense and ultraviolet (UV) and antioxidant protection. Among these, furanocoumarins, known to play a role in plant defense against pathogens and herbivorous insect pests have a negative impact on human health, acting as potent photosensitizers and interacting with medications, effects collectively known as the ‘grapefruit juice effect’. For this reason, the breeding of new citrus varieties suitable for human consumption pursues the reduction in coumarins and furanocoumarins content [2]. Nevertheless, flavonoids and limonoids which are also abundant in citrus species, mostly have beneficial health effects and their enrichment constitutes an important breeding target [3,4]. These metabolites are heterogeneously distributed among citrus species, varieties and fruit tissues contributing to the characteristic bitter flavor trait in citrus [5,6]. Flavonoids and limonoids are widespread in the citrus genus although their distribution varies greatly between organs and tissues (Table 1). Domestication of citrus arising from ancient Southeast Asia to Southern Europe and the Americas has led to particular composition of specialized metabolites among cultivated genotypes within the citrus genus reflecting preferences of human populations ([5] and Table 1). In citrus, bitterness can be described in different ways; ‘immediate’ which is determined by compounds that exist in citrus as tasteless precursors and are metabolized to bitter products and ‘delayed’, associated to newly synthesized (or released from tasteless forms) compounds [7]. Immediate bitterness constitutes a genetic trait subjected to selection contributing to the wide array of current citrus varieties, whereas delayed bitterness is predominantly an environmental response although it might be also subjected to genetic regulation [4,7,8]. In ripe citrus fruits, the balance between bitter flavonoid and limonoid compounds provides the final taste trait ranking mandarins and clementines (derived from *Citrus reticulata* L. Blanco) as ‘sweet’ varieties, sweet orange varieties (cultivars of *Citrus sinensis* L. Osbeck) as bittersweet and grapefruits (*Citrus paradisi* L. Macf.) and sour orange (*Citrus aurantium* L.) as bitter [5,9].

Flavonoids are low-molecular weight polyphenolic specialized metabolites produced via the phenylpropanoid pathway [10]. These metabolites can be further classified into different groups including flavanones, flavones, flavonols and anthocyanins, which are primarily present as glycosyl derivatives. Among these, flavanone *O*-glycosides such as naringin, neohesperidin and poncirin have been associated with citrus bitterness [11]. Limonoids are highly oxygenated triterpenes synthesized through mevalonic acid and methylerythritol 4-phosphate pathways, limonin being the main representative in the *Citrus* genus. In citrus, limonoids comprise around 37 non-glycosylated and 17 glycosylated forms [12]. In general, higher concentrations of limonoid aglycones such as limonin and nomilin are associated to bitterness whereas limonoid glycosides are tasteless [11].

The specific specialized metabolites profile is highly dependent on the background genotype [2,14,15], although their accumulation is also influenced by environmental variables such as abiotic constraints [16], biotic threats [8] as well as developmental processes, especially during fruit maturation [6,9]. The specificity of the flavonoid profile has allowed the use of these metabolites as markers of adulteration in commercial juices [15,17] and the discrimination of several citrus varieties and fruit types [15]. In fruits, the flavonoid and limonoid profile changes over the entire developmental process. For instance, it has been reported that limonin and nomilin levels decline, although low environmental temperature induce fluctuations in their levels [11]. In the ripening process, the most important factor influencing debittering is the induction of limonoid UDP-glucosyl transferases, enzymes that catalyze the glycosylation of limonoid aglycones towards tasteless compounds [6,9]. The concentration of total flavonoids decreases over the ripening process, especially in flavedo, the colored part of the rind [18], but also in pulp tissues [19] in a way associated with the downregulation of chalcone synthase and chalcone isomerase gene expression [20]. Therefore, the specialized metabolite profiles highly depend on fruit development and ripening. In addition to this, depending on the fruit tissue considered, the specialized metabolite blend changes, probably as a result of a different metabolism. In this respect, the edible part of citrus fruits, the endocarp or pulp, is the target tissue in terms of palatability and commercial value, followed by the flavedo that constitutes an important source of specialized metabolites including aroma volatiles, essential oils and waxes, etc. [21,22] with an important involvement in mold and disease resistance [23,24]. The mesocarp, a spongy tissue layer between the flavedo and the pulp, also known as albedo, has traditionally received less attention despite possessing significantly higher levels of flavonoids, as described in several citrus species [25] and its involvement in the regulation of post-harvest peel pitting [26].

Previous research indicates that a delicate balance between flavonoids and limonoids is linked to the development of bitter or sweet taste traits. This makes the assessment of bitterness in citrus a complex task since several metabolites have to be monitored at the same time. In this respect, some studies have focused simultaneously on citrus flavonoids and limonoids [15,27,28,29,30] using high-throughput methods combining ultra-high-performance liquid chromatography (UPLC) and high-resolution mass spectrometers such as hybrid quadrupole/time-of-flight mass analyzers (QqTOF-MS). This combination has proven to be an effective combination for metabolite identification and quantification due to its excellent resolution and high sensitivity [31]. Non-targeted liquid chromatography/electrospray ionization-quadrupole/time-of-flight-mass spectrometry (LC/ESI-QqTOF-MS) metabolite profiling is an efficient technique to profile secondary metabolites in citrus juices with little sample processing (squeezing, centrifuging and filtering). In addition, this technique could be coupled to multivariate analysis as data mining technique to allow separation of different fruit sources and, more importantly, to achieve differentiation of varieties within a particular fruit type group [15]. The aim of this study was to analyze flavonoid and limonoid contents in a non-targeted fashion in order to evaluate the genotype-, tissue- and developmental stage-dependent accumulation of specialized metabolites focusing on the pulp and the albedo [11,32]. To provide a wider view of the differences among citrus genotypes in the biosynthesis and accumulation of specialized metabolites known to contribute to bitterness, representative varieties of the most important citrus fruit types: mandarin, orange and grapefruit were selected, and pulp and albedo samples collected over the developmental process (see Supplementary Figure S1).

## 2. Results and Discussion

### 2.1. Validation of Results, Variable Selection and Annotation of Compounds

**Figure 1:**
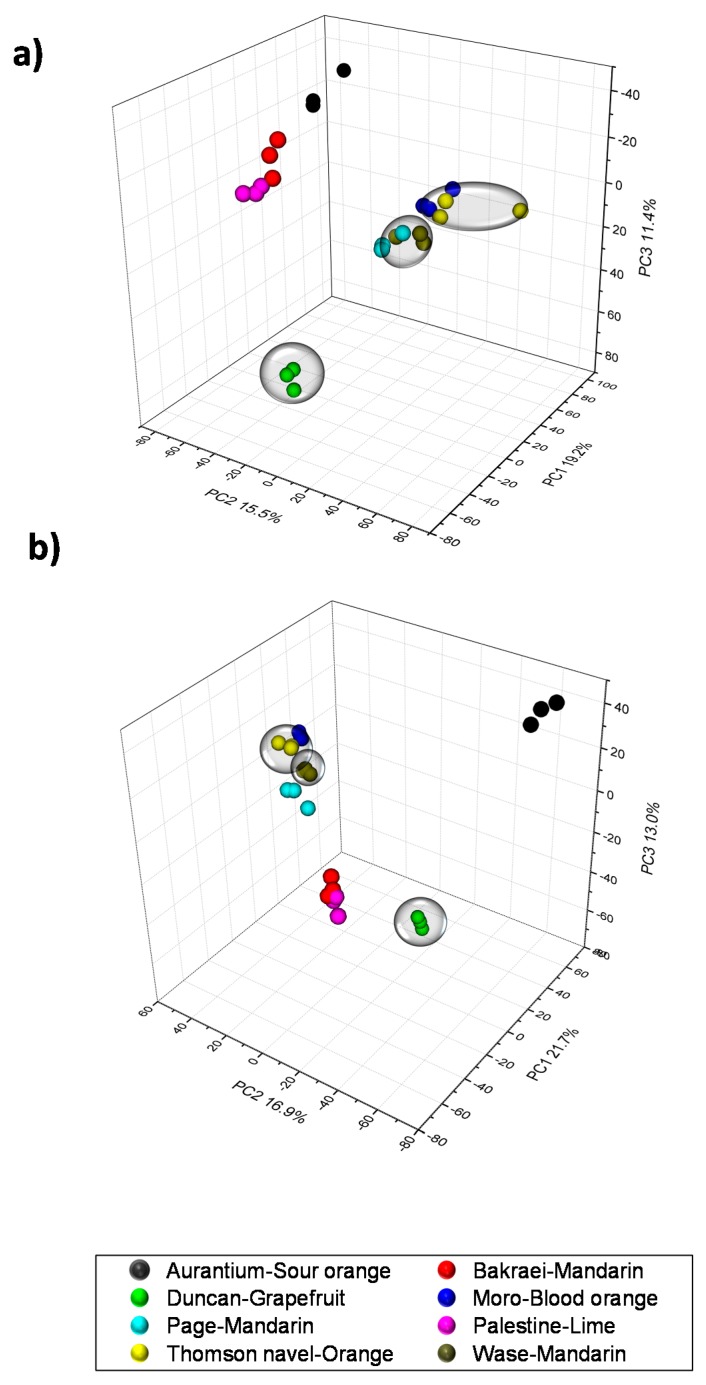
Scores 3D scatter plots after partial least squares-discriminant analysis (PLS-DA) in the pulp of eight citrus genotypes 200 days after full bloom ((**a**): positive electrospray, (**b**): negative electrospray). The genotypes included in subsequent analyses are enclosed in transparent balloons.

As a first approach to identify specialized metabolites discriminating citrus varieties included in this study, a global LC/ESI-QqTOF-MS metabolite profiling was performed using pulp tissues from all eight varieties listed in Supplementary Table S1. Mass chromatographic features were extracted with xcms and subsequently grouped using CAMERA [33]. A matrix containing peak area values was normalized and autoscaled. These data was used to perform a hierarchical cluster analysis (HCA) to depict sample group relationships (Supplementary Figure S2). Results indicated an effective clustering within sample groups: clusters including (1) Wase and Page mandarins; (2) Thomson and Moro oranges (*C. sinensis*); (3) Palestine lime and Bakraei mandarin, resulting from the hybridization of *C. reticulata* and *C. limettoides* (Supplementary Table S1), and two external groups including sour orange (*C. auratium*) and Duncan grapefruit (*C. paradisi*). Data-mining techniques such as multivariate analyses can be applied to the resulting data to separate different fruit sources in citrus in a way reflecting the chemical phenotype and subsequently certain flavor traits [14,15]. To extract significant variables contributing to this classification, a partial least squares-discriminant analysis (PLS-DA) was carried out using sample description in respect of the HCA results to define sample groups. The three first components were selected and the scores presented as a 3D scatter plots (Figure 1). Results indicated an optimal performance of the model showing a cumulative overall cross-validation value of 0.90 and 0.93 in positive and negative electrospray modes, respectively. Taken together, results indicate that the model was accurate enough to differentiate large clusters of fruits in both ionization modes. Three major clusters could be observed in score plots including (1) mandarins and oranges, (2) Bakraei and Palestine and a (3) heterogeneous group including Duncan grapefruit and Aurantium sour orange (see Supplementary Table S1 for a detailed description). Based on this classification, Duncan grapefruit, Thomson navel orange and Wase mandarin, each representing a different degree of bitterness (from bitter to sweet, Duncan grapefruit > Thomson navel orange > Wase mandarin) [5] were selected for a more in-depth analysis.

The selection of mass chromatographic features was carried out using the variable importance for the projection (VIP) value provided by the PLS-DA analysis and the validity of these variables were further confirmed by analysis of variance (ANOVA). As a result, variables showing a VIP value equal or higher than 1.0 ± 0.1 were regarded as important, and the rest discarded. These variables always showed a p-value much lower than the established cutoff value of 0.05 (see Supplementary Table S2). Using this approach, a total of 43 different compounds were identified in citrus pulp (Supplementary Table S2) of which 32 including 19 flavonoids and 13 limonoids were subsequently selected for a deeper examination due to their involvement in immediate and/or delayed bitterness in citrus (Table 2).

**Figure 2:**
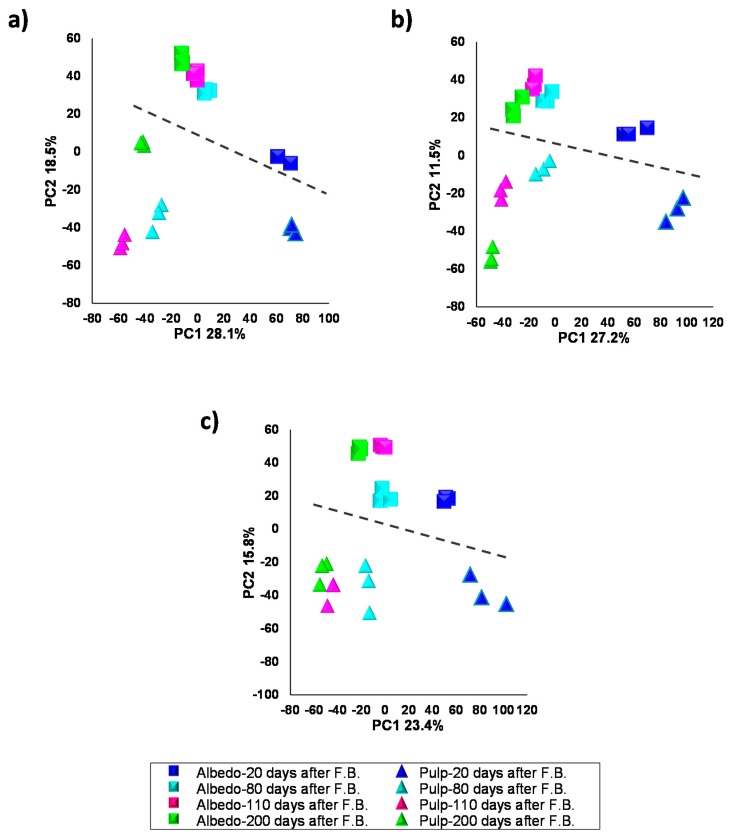
Scores 2D scatter plot after PLS-DA analysis of secondary metabolite profiles from albedo and pulp of Duncan grapefruit (**a**), Wase mandarin (**b**) and Thomson navel orange (**c**) 20, 80, 110 and 200 days after full bloom in negative electrospray ionization (see Supplementary Figure S4 for positive electrospray ionization). The dashed line separates albedo and pulp samples.

In the selected varieties, fruits at four developmental stages (20–200 DAFB) were collected and albedo and pulp tissues isolated, extracted and data analyzed following the procedures described in the Materials and Methods section. PLS-DA was performed using tissue and developmental stage as classification variables (Figure 2). Mass chromatographic analyses were carried out in both ionization modes with a clear better performance of negative electrospray in terms of tissue and developmental stage discrimination (see Figure 2 and Supplementary Figure S4 for positive electrospray). Albedo and pulp showed different specialized metabolite composition and were well resolved over PC2 with variability ranging between 18.5% and 11.5% in all three varieties. These results showed that albedo and pulp, despite being neighboring tissues, had significantly different composition also indicating that cross-contamination during tissue harvesting and processing was negligible. Moreover, variability among developmental stages, resolved over PC1, ranged between 28.1% and 23.4%, indicating that the major source of variability is the developmental stage and not the tissue type (Figure 2).

### 2.2. Characterization of Metabolite Profiles in Albedo and Pulp Tissues

**Figure 3:**
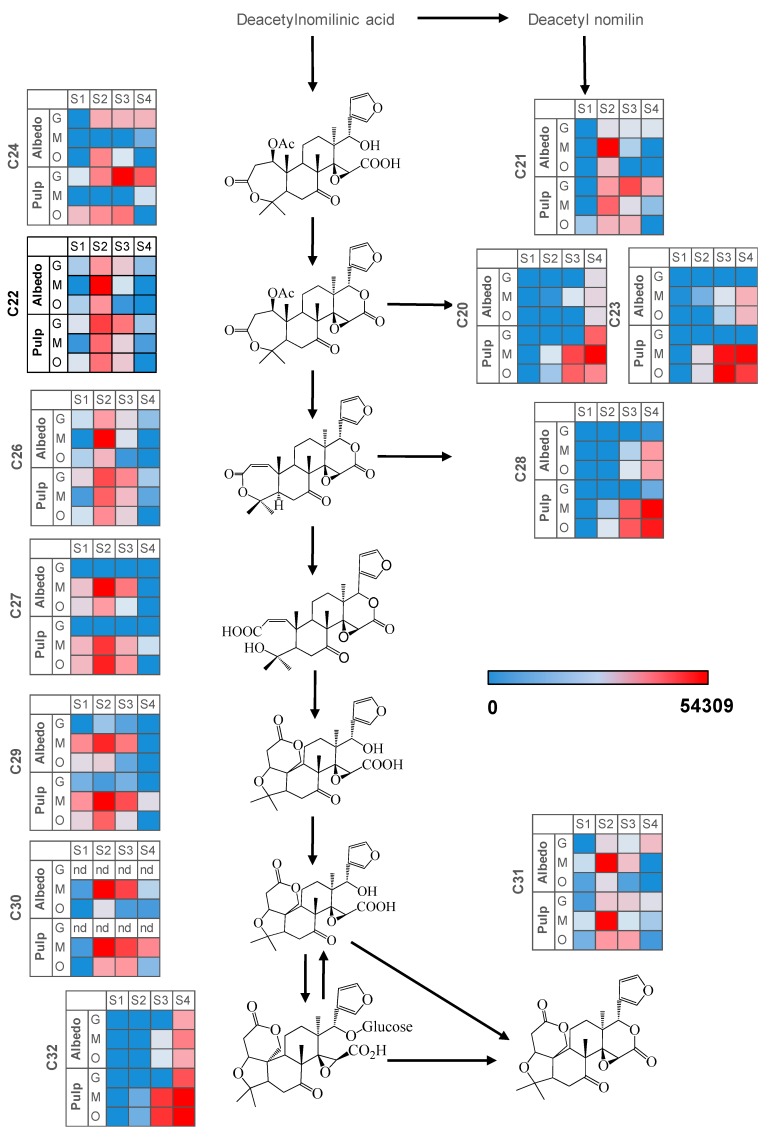
Heatmaps depicting the accumulation of metabolites involved in the limonoid pathway. Normalized metabolite peak area values are expressed as color scale (nd: not detected, S1: 20 days after full bloom, S2: 80 days after full bloom, S3: 110 days after full bloom, S4: 200 days after full bloom, G: Duncan grapefruit, O: Thomson navel orange, M: Wase mandarin). For metabolite identification, please, refer to Table 2.

In all three varieties studied and fruit tissues, most limonoid aglycones (C22, C24, C26, C27, C29, C30 and C31) along with glycoside C21 showed maximum increases at S2 and subsequently reduced their concentration to reach different levels. By contrast, the rest of glycosylated limonoids (C20, C23, C28 and C32) increased their concentration throughout all developmental stages, reaching their maximum concentration at full ripeness (S4). Nevertheless, some deviation from this accumulation pattern was detected regarding tissue and fruit type. For instance, limonoid aglycone C24 could be barely detected in Wase mandarin fruit tissues whereas downstream metabolites such as C22, C26, C27, C29 or C30 showed significantly higher levels in this genotype, suggesting enhanced hydrolase and deacetylase activities rendering C22 and, subsequently, C26 [28]. In orange pulp, C24 showed an accumulation trend up to S3 stage and then dropped to minimal values, whereas in albedo a transient and isolated maximum was recorded at S2. Downstream metabolites were at significantly higher levels compared to grapefruit or mandarin, especially at S2 (Figure 3) suggesting an overall activation of the pathway both supplying the precursor C24 and also transforming it. Interestingly, C24 showed low albeit constant levels in grapefruit albedo after S1 and higher levels in pulp, especially at S3. This trend was associated to reduced levels of C27 and C29 and the absence of C30 in this genotype (Figure 2), consistent with the reported activity of limonin D-ring lactone hydrolase for Duncan grapefruit in comparison to sweet orange varieties [28]. Finally, the bitter limonoid C31 showed a sharp accumulation at S2 in mandarin but it rapidly reduced reaching very low levels at S4, particularly in albedo tissues. Conversely, levels of C31 in grapefruit slightly increased after S1 and remained barely changed thereafter showing the highest values at the full ripe stage S4 (Supplementary Figure S2). For this metabolite, the accumulation profile observed in orange fruit tissues resembled that of mandarin but with significantly lower levels throughout the developmental process. This reversion of C31 levels in albedo and pulp tissues of the two sweeter varieties could be a result of the enhanced expression of limonoid UDP-glucosyl transferase and the use of C31 as a substrate instead of C30. This proposal is consistent with the observed expression of limonoid UDP-glucosyl transferase gene showing a maximum at S3 in mandarin (Supplementary Figure S5). The accumulation profile of the end-products of the pathway: C30, C31 and C32, known to have a role in citrus fruit bitterness, could be clearly associated with the expected taste trait for each citrus variety and tissue (Supplementary Figure S3). Interestingly, the tasteless metabolic precursor of C31, C30, could not be detected in albedo or pulp tissues of Duncan grapefruit whereas its concentration was remarkably high in sweet varieties (mandarin > orange) especially at S4 (Figure 3 and Supplementary Figure S3).

**Figure 4:**
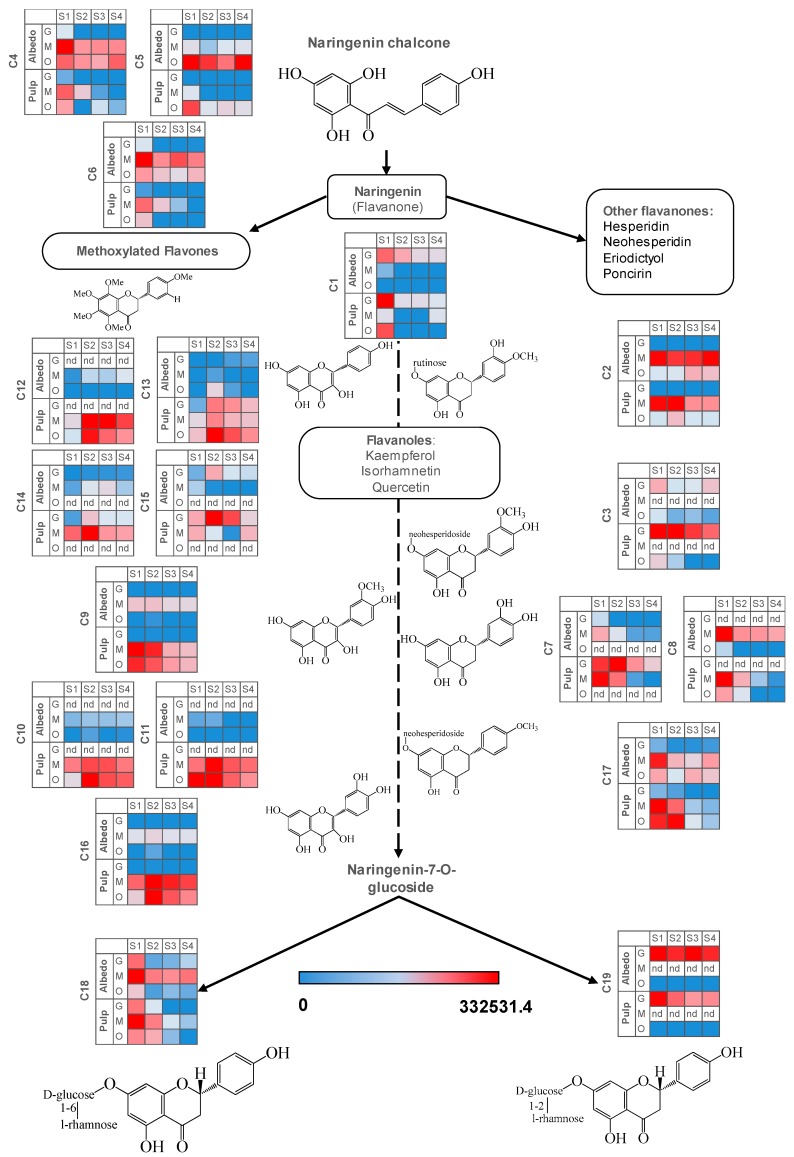
Heatmaps depicting the accumulation of metabolites involved in the flavonoid pathway. Normalized metabolite peak area values are expressed as color scale (nd: not detected, S1: 20 days after full bloom, S2: 80 days after full bloom, S3: 110 days after full bloom, S4: 200 days after full bloom, G: Duncan grapefruit, O: Thomson navel orange, M: Wase mandarin). For metabolite identification, please, refer to Table 2.

The flavonoid pathway in citrus arises from naringenin chalcone rendering different chemical compounds differing in their polyphenolic core structures, namely flavanones, flavones, flavonols, etc and their derivatives. Most flavonoids studied in this work reduced their relative levels in pulp and albedo throughout the developmental process reaching different values depending on the citrus genotype (Figure 4 and Supplementary Figure S3), consistent with the reduced expression of chalcone isomerase gene after S1 (Supplementary Figure S5). For most flavonoid compounds such as C1, C3, C7, C9, C10, C11, C12, C13, C14, C15 and C16 there was a clear partitioning towards the edible part showing significantly increased levels in pulp. For polymethoxylated flavones (C4–C6) the situation was the opposite (Figure 4), showing higher values in albedo. The occurrence of some metabolites was restricted to specific genotypes such as C8, C10, C11 and C12, only present in orange and mandarin, C7, C14 and C15 only present in grapefruit and mandarin and C3 and C19, only present in grapefruit and orange. This could indicate that C13 could be the precursor of the rest of the kaempferol derivatives, particularly C12 and C14, with the addition of a deoxyhexose and a hexose and a caffeoyl moiety, respectively. The precursor of C15 is less clear, although it is possible that a hydroxymethyl glutaryl moiety is attached to a glycosylated kaempferol core structure, constituting C13 also a potential intermediate in the reaction. For isorhamnetin glycosyl derivatives, a clearer picture is devised, being C9 the plausible precursor and C10 and C11 the derivatives whose synthesis is abolished in grapefruit, either the addition of hexose or deoxyhexose. In this case, a different enzyme to that involved in glycosylation of kaempferol derivatives is likely involved. In the other branch, hesperidin (C2) attached to hesperidoside and neohesperidin (C3) attached to neohesperidoside showed nearly opposite trends, the former being highly accumulated in mandarin tissues, especially in albedo, and the latter showing a strong accumulation in pulp tissues in grapefruit, but not detected in mandarin. These results could be partially explained by the genetics of citrus: grapefruit is a hybrid between pummelo (*C. maxima*) and sweet orange (*C. sinensis*). In turn, sweet orange is likely a result of the hybridization of pummelo and the ancestral mandarin (*C. reticulata*). Finally, Wase mandarin, a satsuma mandarin (*C. unshiu*), is thought to be the result of backcrossing a pummelo and a mandarin hybrid [34] as reflected in Supplementary Table S1. The ability to synthesize C12, C14 and C15 is likely an ancestral mandarin trait somehow lost in grapefruit and sweet orange through several backcrosses and selection. Similarly, glycosylation of C9 to render C10 and C11 also seems to be an ancestral mandarin trait, absent in grapefruit. Regarding the synthesis of C3, this is possibly a pummelo trait, lost in satsuma mandarin. Although, the actual carbohydrate positioning of C7 and C8 could not be determined, mass spectrometry data allow us to conclude that both metabolites are two isomeric molecules sharing the eriodictyol core structure: C7 is absent in orange and C8 in grapefruit. In mandarin, despite the two molecules being detected, a tissue specialization in the accumulation of each isomer was observed. A plausible explanation to this observation is that the ability to synthesize both compounds is an ancestral mandarin trait partially inherited by sweet orange and subsequently grapefruit. Indeed, the origin of satsuma mandarin (late admixture mandarins type 3, according to [34]) could explain the presence of both metabolites in this genotype. In grapefruit, metabolite profiles were predominantly composed by neohesperidosides (Figure 4 and Supplementary Figure S3). This is likely a result of the upregulation of genes involved in the transformation of core flavonoid structures rendering bitter 1,2-rhamnosyl derivatives (e.g., naringin) [5,20]. By contrast, sweet varieties such as mandarins and sweet oranges preferentially accumulated *O*-rutinosides as described previously in [15,17] and also reported in this work (Figure 3 and Figure 4). Interestingly, no single compound was found to be absent in both oranges and mandarins, despite their ‘sweet’ trait, reinforcing the admixture origin of all three fruit types [34].

**Figure 5:**
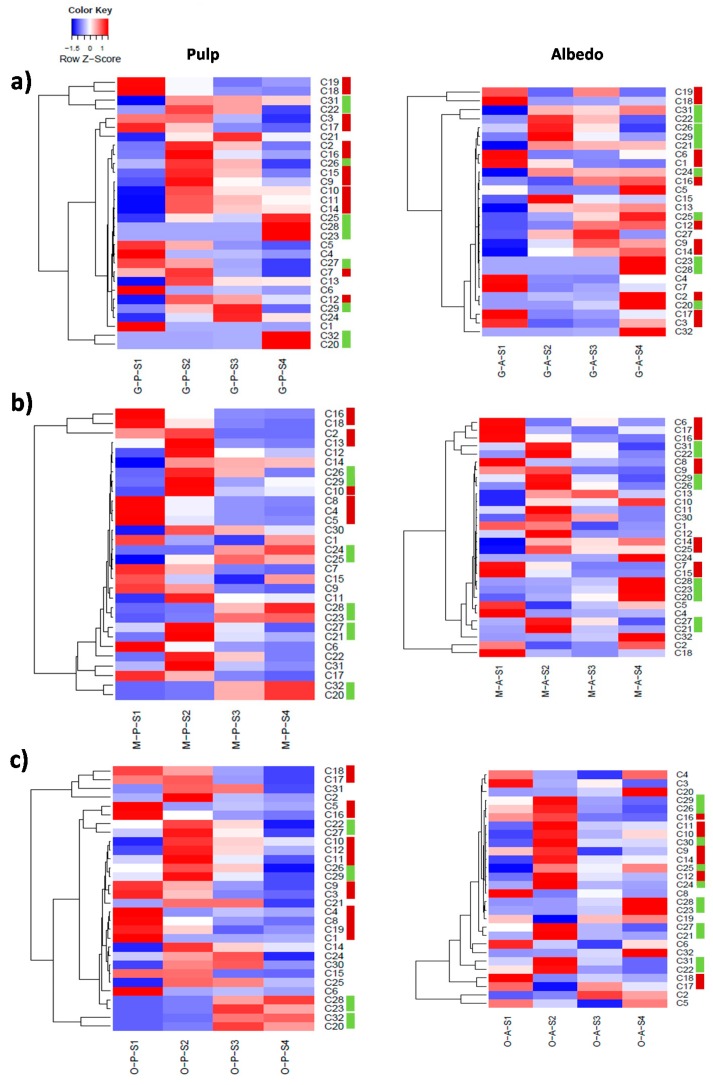
Hierarchical cluster analysis (HCA) depicting the co-regulation of different metabolites involved in the limonoid and flavonoid pathways in Duncan grapefruit (**a**), Wase mandarin (**b**) and Thomson navel orange (**c**) pulp and albedo tissues harvested 20, 80, 110 and 200 days after full bloom (S1 through S4). Relative values are expressed as a heatmap color scale. For metabolite identification, please, refer to Table 2 and Supplementary Table S2. Color bars next to metabolite IDs refer to flavonoids (dark red) and limonoids (light green) in significant clusters.

**Figure 6:**
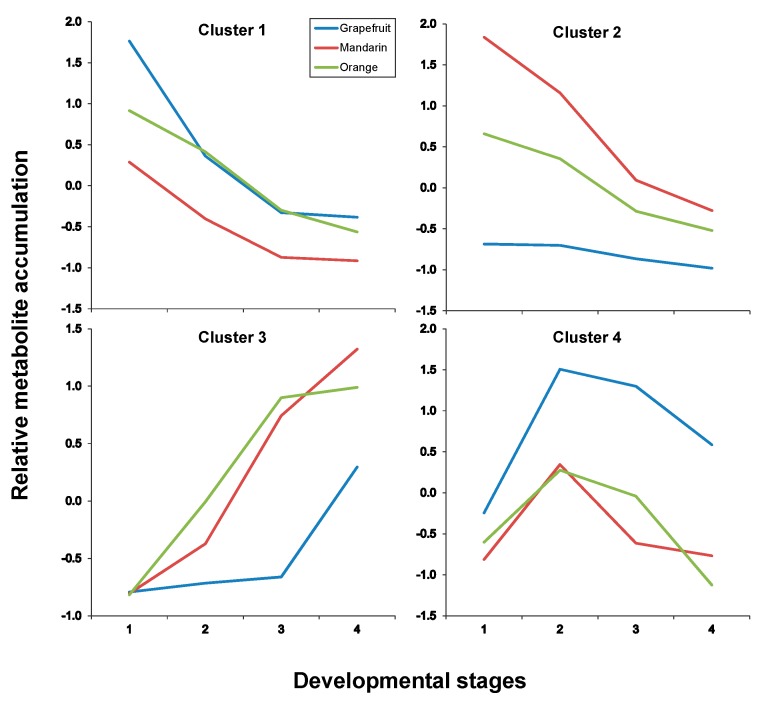
Cluster analysis of metabolite accumulation trends throughout developmental stages after maSigpro analysis (see Materials and Methods in Section 3) in citrus pulp tissues. For developmental stages refer to Figure 3 or Figure 4, and for compounds included in each cluster to Table 3.

**Figure 7:**
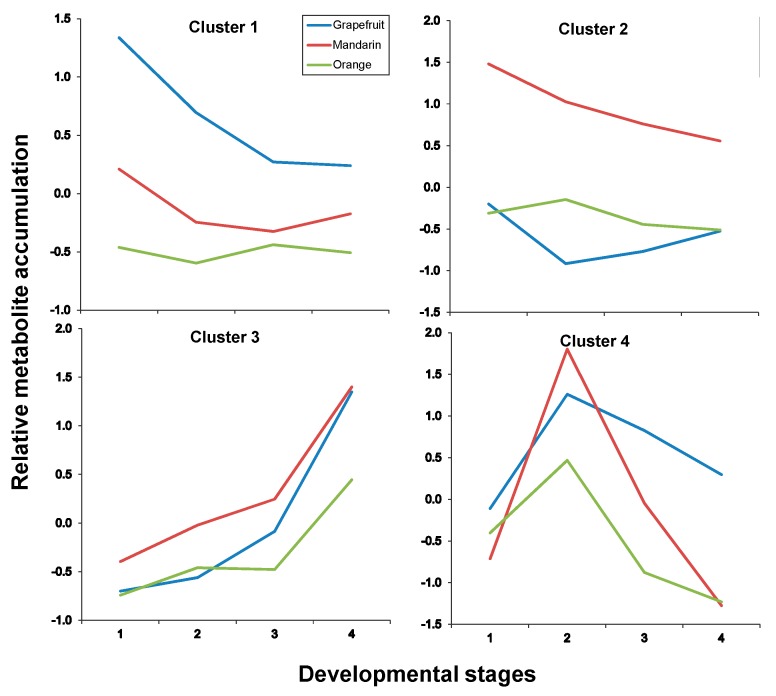
Cluster analysis of metabolite accumulation trends throughout developmental stages after maSigpro analysis (see Materials and Methods in Section 3) in citrus albedo tissues (for developmental stages refer to legends to Figure 3 or Figure 4). For developmental stages refer to Figure 3 or Figure 4, and for compounds included in each cluster to Table 3.

Throughout the entire developmental process, flavonoid concentration, unlike limonoid glycosides, was progressively reduced showing very little values at S4 in both tissues (Figure 4 and Figure 5) except for C14 in albedo (Figure 4, Figure 5, Figure 6 and Figure 7, and Table 3). These observations are consistent with the downregulation of chalcone isomerase gene (Supplementary Figure S5) in pulp tissues. Despite this general trend, differences in the starting and final concentrations were observed among genotypes and tissues (Figure 4). In this sense, typical ‘bitter’ compounds such as C19 showed the highest values in grapefruit followed by sweet orange and presented minimal levels or were not detected in mandarins (Figure 4 and Supplementary Figure S3).

### 2.3. Fruit Ripening: Co-Regulation of Specialized Metabolites in Citrus Fruit Tissues

The fruit-ripening process involves the coordinated regulation of several metabolites including sugars and acids and also specialized metabolites such as flavonoids and limonoids. To investigate this aspect, first a HCA analysis to group metabolites showing similar accumulation patterns over the developmental process within genotypes and tissues (Figure 5) and, subsequently, conserved metabolite accumulation profiles among genotypes (Figure 6 and Figure 7), were performed.

In both albedo and pulp, flavonoids decreased their concentration with ripening showing different trends: a maximum at S1 and decreasing afterwards. Conversely, limonoids and especially limonoid glycosides accumulated with ripening including limonoid glycosides C28 and C32. Interestingly, apart from grapefruit that showed C27 peaking at S1 no other genotype showed such an early induction of the limonoid pathway in pulp tissues, followed by C26 that peaked at S2 to decrease thereafter in all three varieties (Figure 3 and Figure 5). Therefore, limonoid glycoside accumulation is likely a ripening-associated trait in citrus.

The behavior of flavonoids and limonoids in pulp tissues was almost identical among citrus varieties throughout the developmental process, despite differences in relative levels and the specific accumulation trends summarized above. However, when comparing metabolite trends among varieties in albedo a great divergence was found between grapefruit, with a higher number of compounds showing a maximum at S4, including a limonoid (C31), several limonoid glycosides (C20, C23, C25, C28 and C32) and a number of flavonoids (C2, C5 and the kaempferol derivatives C12, C13 and C14) and orange and mandarin where only limonoid glycosides showed a clear maximum at S4. These results revealed an apparent uncoupling of the metabolism of specialized metabolites in pulp and albedo in grapefruit whereas in orange and mandarin these showed a parallel behavior (Figure 5).

The co-regulation of flavonoids varied among genotypes. Except for orange pulp, precursor C1 showed poor connection to the rest of flavonoids detected (Figure 5), suggesting that their regulation took place at later steps. Compounds C18 and C19, known for their involvement in bitterness in citrus, clustered tightly in grapefruit fruit tissues (albedo and pulp), whereas C18 clustered with C16 and with C17 in mandarin and orange, respectively (Figure 5). C1 glycosylation on 7′-OH catalyzed by 7-glycosyl transferase followed by the addition of a rhamnose moiety catalyzed by 1,6-rhamnosyl transferase renders non-bitter C18 whereas the accumulation of bitter C19 [5] is the result of the reaction catalyzed by 1,2-rhamnosyl transferase, which seems to be inactive in mandarin (Figure 4). This is consistent with the observed metabolic connectivity of C18 with C16 in mandarin and with C17, a 7-*O*-neohesperidoside, in orange, since 1,2-rhamnosyl transferase is involved in its biosynthesis [5].

In both tissues, four clusters were identified as a result of maSigPro analysis, each depicting an accumulation trend over the developmental process for the varieties included in this study. In pulp tissues, Clusters 1 and 2 grouped all metabolites that showed a decreasing pattern over time but with significant differences in the decay trend; Cluster 3 grouped all metabolites that increased their concentration with time and Cluster 4 grouped metabolites showing a transient accumulation followed by decay or stabilization (Figure 6 and Supplementary Table S7 for p-values and correlation values). As observed throughout different analyses and representations, metabolite trends followed a compound class-specific behavior. Hence, flavonoids identified in citrus fruits were grouped following two distinct decreasing trends: Cluster 1 including compounds C1, C7, C8, C13, C17 and C19 that showed a decreasing trend arising from S1 (Figure 6 and Figure 7, and Table 3) and exhibited higher levels in grapefruit (and in orange albedo) than in the rest of varieties, and Cluster 2 that grouped compounds C2, C3, C4, C5, C6 and C9 whose depletion was more pronounced in pulp but presented a shallower trend in albedo. Moreover, these compounds showed significantly higher levels in mandarin throughout the entire developmental process followed by orange and grapefruit, suggesting that these compounds are not likely related to bitterness in citrus since their abundance was oppositely associated to the expected taste trait in the selected varieties (mandarin > orange > grapefruit). Besides flavonoids, Cluster 2 also comprised limonoids C29 and C30 in pulp and only C30 in albedo. These remained almost unchanged in Duncan grapefruit fruit tissues and decreased sharply in Wase mandarin pulp showing a more moderate reduction in the edible part of Thomson orange. In the two sweet varieties, the decreasing trend was evident in pulp tissues whereas in albedo it was less pronounced nearly showing a flat trend over the entire developmental period (Figure 7 and Supplementary Table S8 for *p*-values and correlation values). Cluster 3 included metabolites whose concentration increased with fruit maturity: C14 and three glycosylated limonoids: C23, C28 and C32. It is interesting to note that while the behavior of these metabolites in pulp tissues of Wase mandarin and Thomson orange was almost the same, they remained almost unchanged at low levels in Duncan grapefruit for most of the developmental period showing a sharp accumulation at S4. Conversely, the same compounds plus C18 showed a parallel behavior in mandarin and grapefruit reaching similar final values whereas orange followed a slightly different behavior. Finally, Cluster 4 comprised all metabolites that showed a transient increase at S2 and decreased thereafter reaching different final levels at S4. This group included the flavonoid derivative C15 and the limonoids: C22, C24, C25, C26, C31 (Table 3). These compounds showed nearly parallel and identical trends in orange and grapefruit and also comparing both fruit tissues (Figure 6 and Figure 7) whereas in mandarin the tendencies between albedo and pulp were clearly different. The transient metabolite accumulation at S2 was more pronounced in mandarin albedo than in pulp and so it was their decay afterwards. It is remarkable the higher levels of typical bitter compounds such as C22, C26 and C31 found in grapefruit tissues at S4, in line with the expected characteristic taste trait of this genotype. Moreover, the occurrence of kaempferol caffeoyl conjugate in Cluster 3 along with other metabolites showing an identical accumulation tendency suggests their uncoupling from its potential precursor C13 in pulp (Cluster 1) and from the hydroxymethylglutaryl kaempferol derivative (C15) in albedo (Cluster 1).

## 3. Materials and Methods

### 3.1. Plant Material and Sample Preparation

Citrus fruits from different genotypes and varieties (see Supplementary Table S1) were harvested from adult trees at the germplasm bank (Citrus Research Institute, Ramsar, Iran). Trees from all varieties included in this study were grafted onto sour orange (*Citrus aurantium* L.) rootstock. Selected harvesting dates were 20, 80, 110 and 200 days after full bloom. From each replicate tree (*n* = 3), a minimum of four fruits, one from each direction on the tree, were collected and pooled. For every sample, albedo and pulp tissues were accurately separated avoiding cross-contamination and immediately frozen in liquid nitrogen. Frozen tissues were subsequently subjected to freeze drying and then ground to fine powder and kept at −20 °C until analyses. For a complete scheme of the experiments performed and the analyses carried out, please refer to Supplementary Figure S1.

### 3.2. Extraction of Samples for Chromatographic Analyses

About 5 mg of freeze-dried ground pulp or albedo tissue was weighted and 500 µL of 80% aqueous methanol (LC/MS grade, Merck, Darmstadt, Germany) spiked with biochanin A to a 1 mg·L^−1^ concentration as internal standard, was immediately added. Tubes containing samples were incubated in an ultrasonic bath for 10 min at room temperature. Afterwards, extracts were centrifuged at 10,000 rpm for 10 min at 4 °C and supernatants recovered and filtered through disposable 0.22 µm PTFE membrane syringe filters. The filtrate was collected in screw-cap amber glass vials fitted with 300 µL-glass inserts that were used for chromatographic analyses.

### 3.3. Chromatographic and QqTOF-MS Conditions

Sample extracts were separated by reversed phase LC using LC/MS-degree acetonitrile (B) and water (A), both supplemented with formic acid to a concentration of 0.1% (*v*/*v*) as solvents. Separations were carried out on a C18 column (Luna Omega Polar C18, 100 × 2.1 mm, 1.6 µm particle size, Phenomenex España, S.L.U., Madrid, Spain). The separation module, a Waters Acquity SDS was operated in gradient mode for 25 min as follows: 0–2 min 95:5 (A:B) followed by an increase in B from 5 to 95 in the following 17 min (2.01–17.00 min), thereafter returning to initial conditions (17.01–20.00 min) that were maintained for 5 min for column reconditioning. During mass chromatographic data acquisition, flow rate was maintained at 300 μL·min^−1^ and column temperature at 40 °C. Column eluates were introduced into a QqTOF-MS (QTOF Premier, Micromass Ltd., Manchester, UK) through orthogonal electrospray ionization (ESI) source operated in positive and negative ionization modes. Nitrogen was used as the nebulization as well as the desolvation gas and working flows were set at 60 and 800 L·h^−1^, respectively. Source block temperature was kept at 120 °C and desolvation gas at 350 °C. Capillary, cone, and extractor voltages were set at 3.5 kV, 30 eV, and 3 eV, respectively. Before analyses, the QqTOF-MS was calibrated by infusing a mixture of NaOH and HCOOH at a flow rate of 25 μL·min^−1^. After calibration, the average mass error was less than 5 ppm. Samples were analyzed in both negative and positive ionization modes. To attain this, two acquisition functions were set: function 1 used collision-induced dissociation energy (CID) of 2 eV and function 2, CID energy ramped from 4 to 65 eV. Additionally, to ensure accurate mass measurements, during acquisitions, a 1 mg·L^−1^ solution of Leu-enkephalin ([M+H]^+^ = 556.2771, [M−H]^−^ = 554.2614) was continuously infused as a lockmass reference through the dual LockSpray^TM^ source of QTOF Premier and acquired in function 3. All measurements were acquired under continuous mode in the 50–1000 amu range, scan duration was set at 1.0 s, and interscan delay was set at 0.1 s. The automatic processing of mass spectra (centroidization of continuous mass data using the lockmass reference) upon acquisition was set.

### 3.4. Mass Spectrometry (MS) Data Processing, Statistical Analyses and Compound Identification

Raw mass chromatograms were converted to netCDF prior to xcms processing [35]. Extracted mass chromatographic data were corrected to internal standard intensity and actual sample weight prior to any statistical analysis (Supplementary Tables S3–S6). Autoscaling was performed variable-wise for the entire dataset by substracting the average value for each variable throughout individual samples and dividing by the standard deviation, as described in [36].

Hierarchical cluster analysis (HCA) was performed with the R package pvclust on the same autoscaled data using Euclidean distance as the distance metrics and average as the clustering method. To identify the variables contributing to this classification, a partial least squares-discriminant analysis (PLS-DA) was performed with SIMCA-P+ 11.0 (Umetrics, Umea, Sweden) using the same autoscaled peak area values setting sample class defined by the HCA results. The number of potential variables contributing to the classification was selected based on variable importance in the projection (VIP) values equal or higher than 1.0 ± 0.1. Confirmation of selected variables was achieved by means of one-way ANOVA using genotype as factor and setting a *p*-value cutoff value of 0.05. Subsequent annotation of compounds was carried out following a step-wise procedure: (1) identification and annotation of precursor ions, (2) annotation of fragment ions and (3) database search (metlin, Human Metabolome Database and Massbank). Metabolite hits meeting the selection criteria (mz value of precursor ion falling within 0.025 amu mass difference and exact match of predominant product ions) were considered to be positively identified. When available, co-injection with pure standards was carried out to undeniably identify metabolites (annotation level a). If no standard is commercially available, but the database search met the selection criteria detailed in step 2, metabolites were annotated following their most plausible candidate (annotation level b). Finally, when product ions partially matched database MS/MS data for each precursor ion hit, metabolites were tentatively annotated (annotation level c). For metabolites for which no standard was commercially available, co-injection of a similar compound allowed the confirmation of precursor-to-product transitions, therefore metabolites were labeled as a,b annotation (Supplementary Table S2).

Heatmaps were built using the gplots R package using autoscaled peak area values of pseudomolecular ions ([M+H]^+^ or [M−H]^−^) throughout all 24 observations (2 tissues × 3 cultivars × 4 developmental stages).

To investigate significantly different accumulation profiles among citrus varieties throughout the developmental process, maSigPro R package [37] was used. This software, initially designed to analyze time-series gene expression data in order to identify genes that show different gene expression profiles across analytical groups over the experimental period, performs a two-regression step approach. First, the maSigPro algorithm uses least-squares and ANOVA to identify differentially expressed variables (metabolites). Afterwards and following an iterative regression-based variable selection strategy, the software selects those variables showing a significant *p*-value conveniently adjusted to reduce type I risk.

## 4. Conclusions

In citrus, the characteristic bitter taste found in certain varieties is the result of a blend of specialized compounds namely flavonoids and limonoids. In this work, metabolite profiles were specific of each citrus genotype and also reflected the characteristic chemical signature associated to particular flavor traits, thus confirming previous reports [15]. Within each variety, the main source of metabolite variability was developmental stage-dependent despite certain degree of variation could be found between neighboring fruit tissues albedo and pulp. During the developmental process, flavonoid concentration decreased whereas limonoids and especially limonoid glycosides accumulate in fruit tissues reaching both types of compounds’ different levels at the full ripe stage. This tendency was compound-class associated with no divergence among citrus varieties and fruit tissues. The differences found between tissues were mainly associated with different levels of specialized metabolites. In this respect, throughout the entire developmental process albedo showed a higher accumulation of polymethoxylated flavones and a lower content in the rest of flavonoids and glycosylated limonoids compared to pulp tissues. To sum up, the specialized compound composition at the full ripe stage and, therefore, the resulting flavor output is linked to the different rate of compound transformation existing among genotypes which seems to be genetically regulated.

## Figures and Tables

**Table 1:** Specialized metabolites known to contribute to bitterness (adapted from [5,13]).

Compound Class	Compound Name	Taste Trait
Flavanones	Naringin (*O*-neohesperidoside)	Bitter
	Narirutin (*O*-rutinoside)	Tasteless
	Diosmin (*O*-rutinoside)	Tasteless
	Neodiosmin (*O*-neohesperidoside)	Bitter
Flavones	Tangeretin	Bitter
	Nobiletin	Bitter
	Sinensetin	Bitter
Flavonols	Rutin	Tasteless
Limonoid aglycones	Limonin D-ring lactone	Tasteless
	Nomilin	Bitter
	Limonin A-ring lactone	Bitter
Limonoid glycosides	Limonin D-ring glycoside	Tasteless

**Table 2:** Selection of annotated compounds (Supplementary Table S1) involved in flavonoid and limonoid biosynthesis.

	Compound	Chemical Formula	Quantifier Ion ESI+	Annotation Positive	Quantifier Ion ESI−	Annotation Negative	Retention Time (min)
***Flavonoids***							
**C1**	Naringenin	C_15_H_12_O_5_	273.07	[M+H]+	271.06	[M−H]−	9.05
**C2**	Hesperidin	C_28_H_34_O_15_	303.09	[M-hesperidoside]+	301.07	[M-hesperidoside]−	6.92
**C3**	Neohesperidin	C_28_H_34_O_15_	303.09	[M-neohesperidoside]+	301.07	[M-hesperidoside]−	7.7
**C4**	Isosinensetin	C_20_H_20_O_7_	373.13	[M+H]+	nd	nd	9.75
**C5**	Sinensetin	C_20_H_20_O_7_	373.13	[M+H]+	nd	nd	10.4
**C6**	Tangeretin	C_20_H_20_O_7_	373.13	[M+H]+	nd	nd	11.6
**C7**	Eriodictyol rutinoside #1	C_27_H_32_O_15_	597.17	[M+H]+	595.17	[M−H]−	5.31
**C8**	Eriodictyol rutinoside #2	C_27_H_32_O_15_	597.17	[M+H]+	595.175	[M−H]−	6.14
**C9**	Isorhamnetin-3-*O*-rutinoside	C_28_H_32_O_16_	625.19	[M+H]+	623.18	[M−H]−	6.71
**C10**	Isorhamnetin rutinoside hexoside	C_34_H_42_O_21_	787.22	[M+H]+	785.21	[M−H]−	5.36
**C11**	Isorhamnetin rutinoside deoxyhexoside	C_34_H_42_O_20_	771.23	[M+H]+	769.22	[M−H]−	6.07
**C12**	Kaempferol diDeoxyhexoside hexoside	C_33_H_40_O_19_	595.16	[M-Hexose]+	739.21	[M−H]−	6.03
**C13**	Kaempferol Deoxyhexoside hexoside	C_27_H_30_O_15_	595.17	[M+H]+	nd	nd	6.62
**C14**	Kaempferol Caffeoyl Hexoside Deoxyhexoside	C_36_H_36_O_18_	757.23	[M+H]+	755.21	[M−H]−	5.28
**C15**	Kaempferol hidoxymethyl glutaryl (HMG)-glucoside *tentative*	C_27_H_28_O_15_	593.15	[M+H]+	591.13	[M−H]−	7.12
**C16**	Quercetin hexoside rutinoside	C_33_H_40_O_21_	773.21	[M+H]+	771.26	[M−H]−	5.01
**C17**	Poncirin	C_28_H_34_O_14_	595.20	[M+H]+	593.19	[M−H]−	7.94
**C18**	Narirutin	C_27_H_32_O_14_	581.18	[M+H]+	579.17	[M−H]−	5.53
**C19**	Naringin	C_27_H_32_O_14_	581.18	[M+H]+	579.17	[M−H]−	6.65
***Limonoids***							
**C20**	Deacetyl Nomilinic acid glycoside *tentative*	C_32_H_46_O_15_	nd	nd	669.27	[M−H]−	6.55
**C21**	Deacetyl Nomilin glycoside	C_32_H_44_O_14_	nd	nd	651.27	[M−H]−	6.96
**C22**	Nomilin	C_28_H_34_O_9_	515.23	[M+H]+	513.21	[M−H]−	11.14
**C23**	Nomilinic acid glycoside	C_34_H_48_O_16_	735.29	[M+Na]+	711.29	[M−H]−	7.37
**C24**	Nomilin A-ring lactone	C_28_H_36_O_10_	533.24	[M+H]+	531.23	[M−H]−	10.53
**C25**	Nomilin glycoside tentative	C_34_H_44_O_14_	nd	nd	675.27	[M−H]−	9.51
**C26**	Obacunone	C_26_H_30_O_7_	455.23	[M+H]+	nd	nd	11.16
**C27**	Obacunoic acid	C_26_H_32_O_8_	473.21	[M+H]+	471.20	[M−H]−	10.20
**C28**	Obacunone glycoside	C_32_H_42_O_13_	nd	nd	633.25	[M−H]−	7.71
**C29**	Ichangin	C_26_H_32_O_9_	489.21	[M+H]+	487.19	[M−H]−	9.63
**C30**	Limonoate A-ring lactone	C_26_H_32_O_9_	489.21	[M+H]+	487.19	[M−H]−	9.81
**C31**	Limonin	C_26_H_30_O_8_	471.20	[M+H]+	469.18	[M−H]−	10.57
**C32**	Limonin 17-β-d-glucopyranoside	C_32_H_42_O_14_	471.20	[M-Hexose]+	649.25	[M−H]−	6.43

nd = not detected.

**Table 3:** Cluster analysis of metabolite accumulation trends throughout developmental stages after maSigpro analysis (refers to Figure 6 and Figure 7).

Pulp	
Cluster #	Metabolites *
Cluster 1	C1, C7, C8, C13, C17, C19
Cluster 2	C2, C3, C4, C5, C6, C9, C27, C29, C30
Cluster 3	C14, C20, C23, C28, C32
Cluster 4	C15, C21, C22, C24, C25, C26, C31
**Albedo**	
Cluster #	Metabolites
Cluster 1	C1, C15, C17, C19
Cluster 2	C2, C3, C6, C7, C8, C12, C30
Cluster 3	C14, C18, C23, C25, C28, C32
Cluster 4	C21, C22, C26, C31

* For metabolite annotations refer to Table 2 and Supplementary Table S2.

## Supplementary Materials

Supplementary materials can be found at https://www.mdpi.com/1422-0067/20/5/1245/s1. Figure S1. Experimental design. Figure S2. Hierarchical cluster analysis (HCA) of citrus pulp samples using autoscaled metabolite profiling data in positive (a) and negative (b) electrospray ionization modes. O, Wase mandarin; T, Thomson sweet orange; G, Duncan grapefruit; M, Moro sweet orange; N, sour orange; L, Palestine lime; B, Bakraei lime; P, Page mandarin. Figure S3. Levels of secondary metabolites reported to have a role in bitterness in citrus (Table 1). Bars labeled with asterisks (*) indicate varieties selected for subsequent studies. Figure S4. Scores 2D scatter plot after PLS-DA analysis of specialized metabolite profiles from albedo and pulp of Duncan grapefruit (a), Wase mandarin (b) and Thomson navel orange 20, 80, 110 and 200 days after full bloom in positive electrospray ionization. The dashed line separates albedo and pulp samples. Figure S5. Expression levels of genes involved in biosynthesis of flavonoids and limonoids. See Supplementary Material and Methods for details on total RNA extraction and quantitative real-time polymerase chain reaction (qRT-PCR) analyses. Table S1. List of genotypes included in this study. Table S2. Identification of metabolites as extracted from PLS-DA. VIP: Variable Importance; ID level: (a) co-injection with standard, (b) mass spectrometry data matches those in databases (metlin or Human Metabolome Database), (c) tentatively annotated based on mass spectrometry data and database search (See Material and Methods section for details). (*) *p*-value after one-way ANOVA using genotype as factor. Table S3. Normalized pulp metabolite profiles in positive electrospray mode. Table S4. Normalized albedo metabolite profiles in positive electrospray mode. Table S5. Normalized pulp metabolite profiles in negative electrospray mode. Table S6. Normalized albedo metabolite profiles in negative electrospray mode. Table S7. Statistical values of specialized metabolites showing significantly different profiles genotype- and developmental stage-wise in fruit pulp (Corresponding to Figure 6). Table S8. Statistical values of specialized metabolites showing significantly different profiles genotype- and developmental stage-wise in fruit albedo (Corresponding to Figure 7). Supplementary Materials and Methods S1. Total RNA isolation, cDNA synthesis and qRT-PCR analyses.
