# Supplementary material for: Developmental Stage- and Genotype-Dependent Regulation of Specialized Metabolite Accumulation in Fruit Tissues of Different Citrus Varieties
Source: Int J Mol Sci. 2019 Mar 12;20(5):1245. doi: 10.3390/ijms20051245 (PMC6429498; doi:10.3390/ijms20051245)

Developmental stage- and genotype-dependent regulation of specialized metabolite accumulation in fruit tissues of different citrus varieties

Roya Nadi, Behrouz Golein, Aurelio Gómez-Cadenas and Vicent Arbona

Supplementary Material

Supplementary Figure S1. Experimental design.

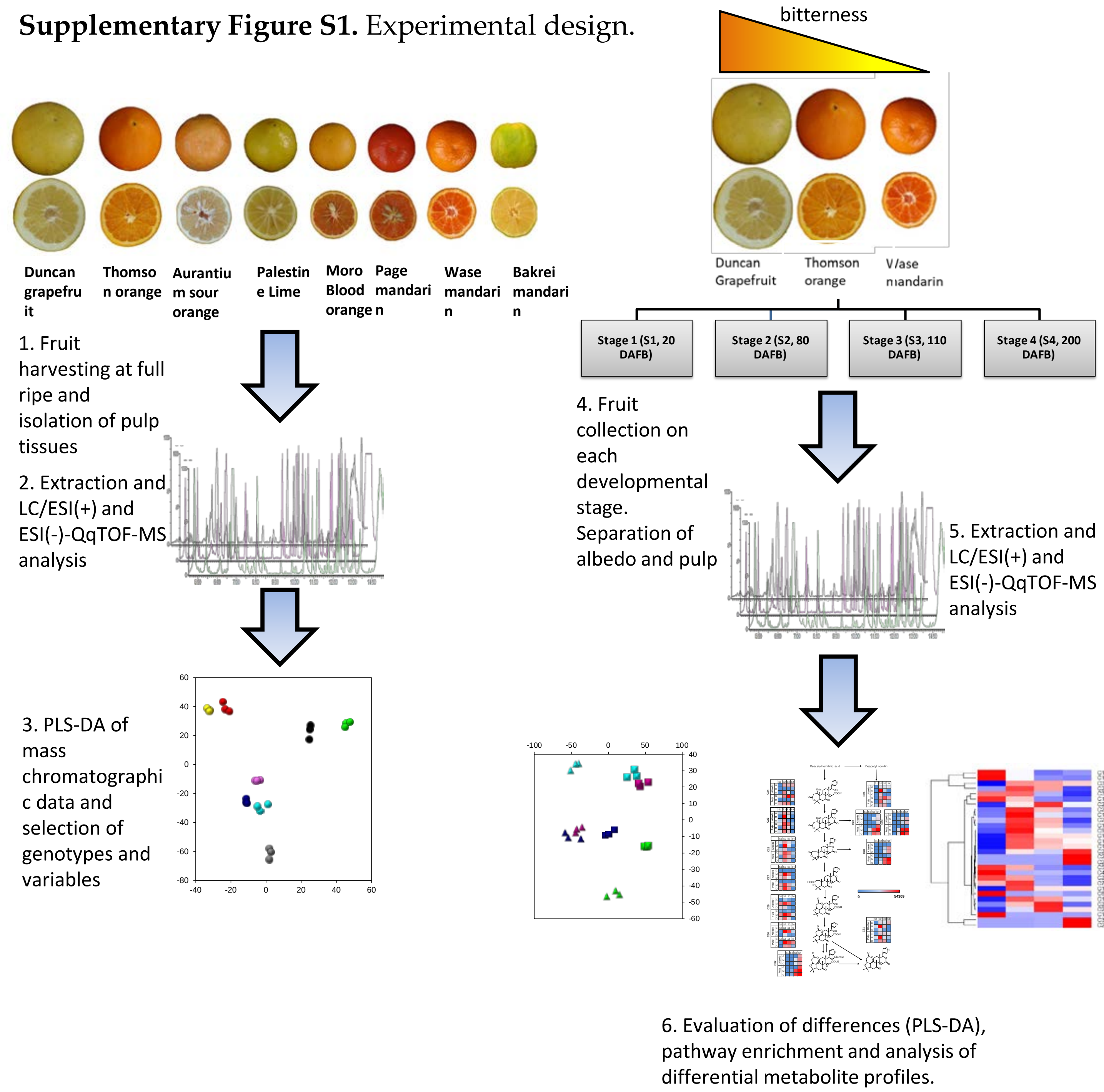

Supplementary Figure S2. Hierarchical cluster analysis (HCA) of citrus pulp samples using autoscaled metabolite profiling data in positive a) and negative b) electrospray ionization modes. O, Wase mandarin; T, Thomson sweet orange; G, Duncan grapefruit; M, Moro sweet orange; N, sour orange; L, Palestine lime; B, Bakraei lime; P, Page mandarin.

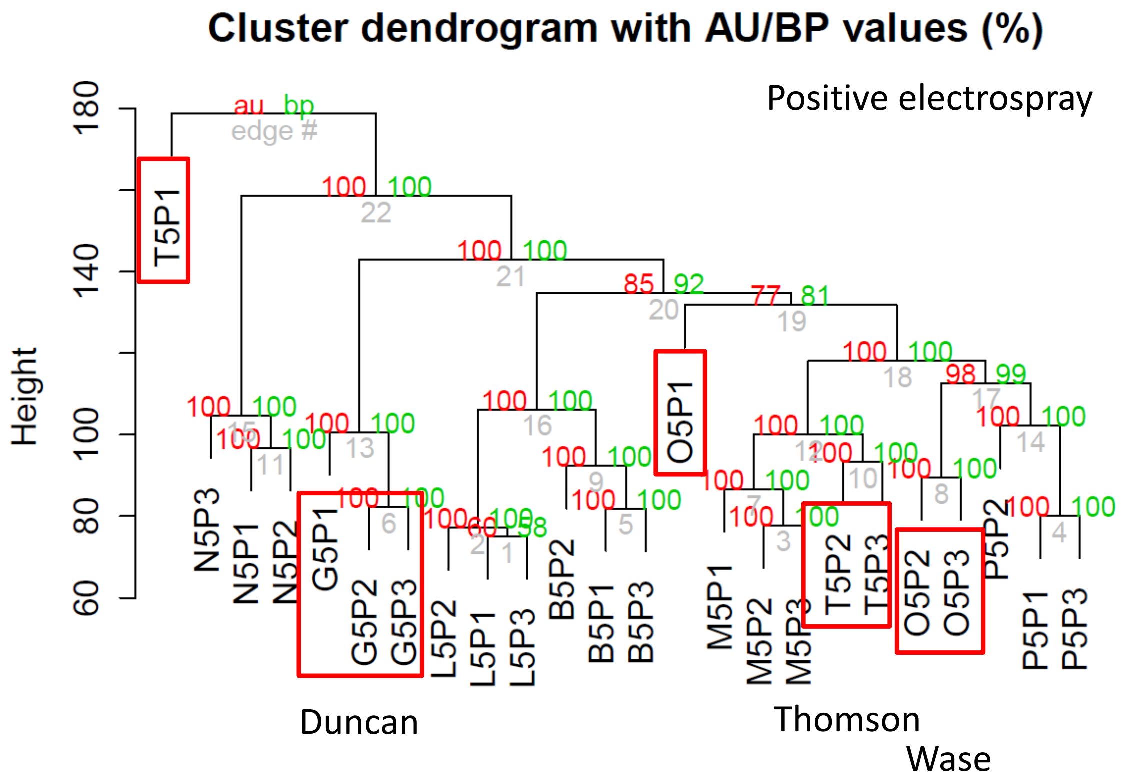

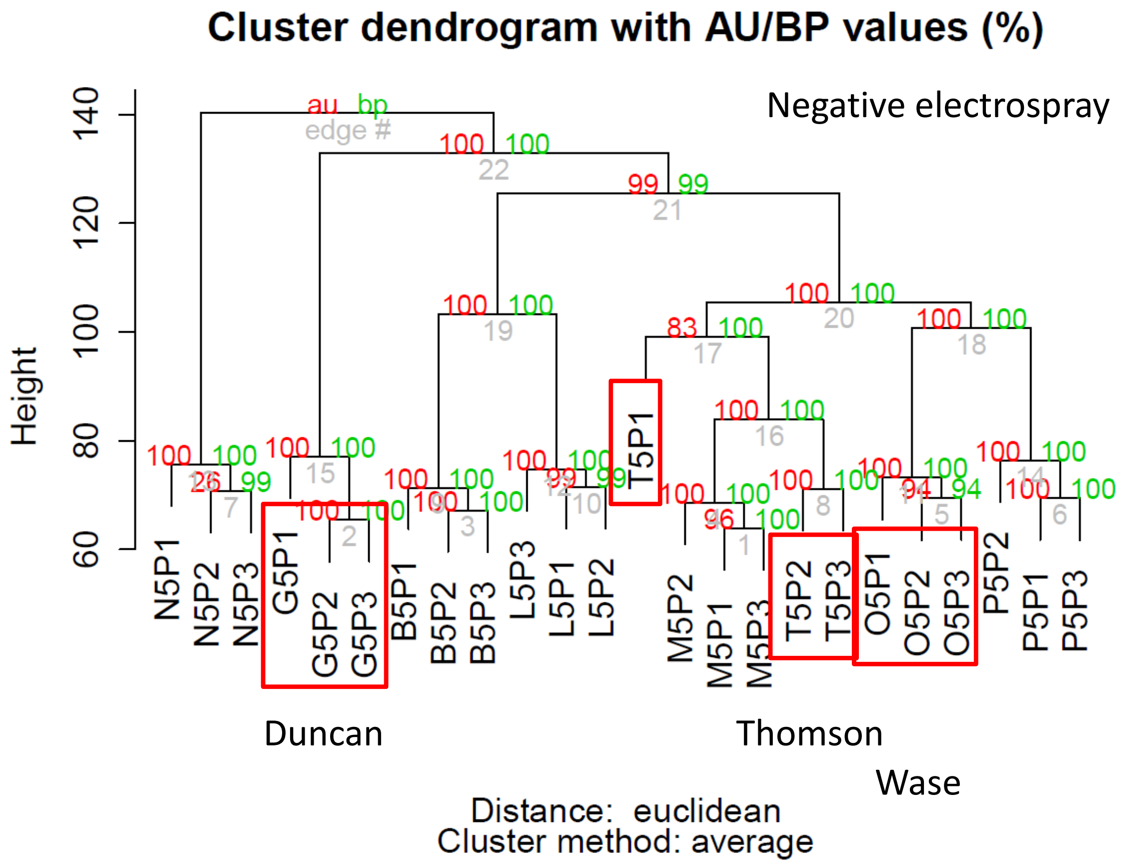

Supplementary Figure S3. Levels of secondary metabolites reported to have a role in bitterness in citrus (Table 1). Bars labeled with asterisks (*) indicate varieties selected for subsequent studies.

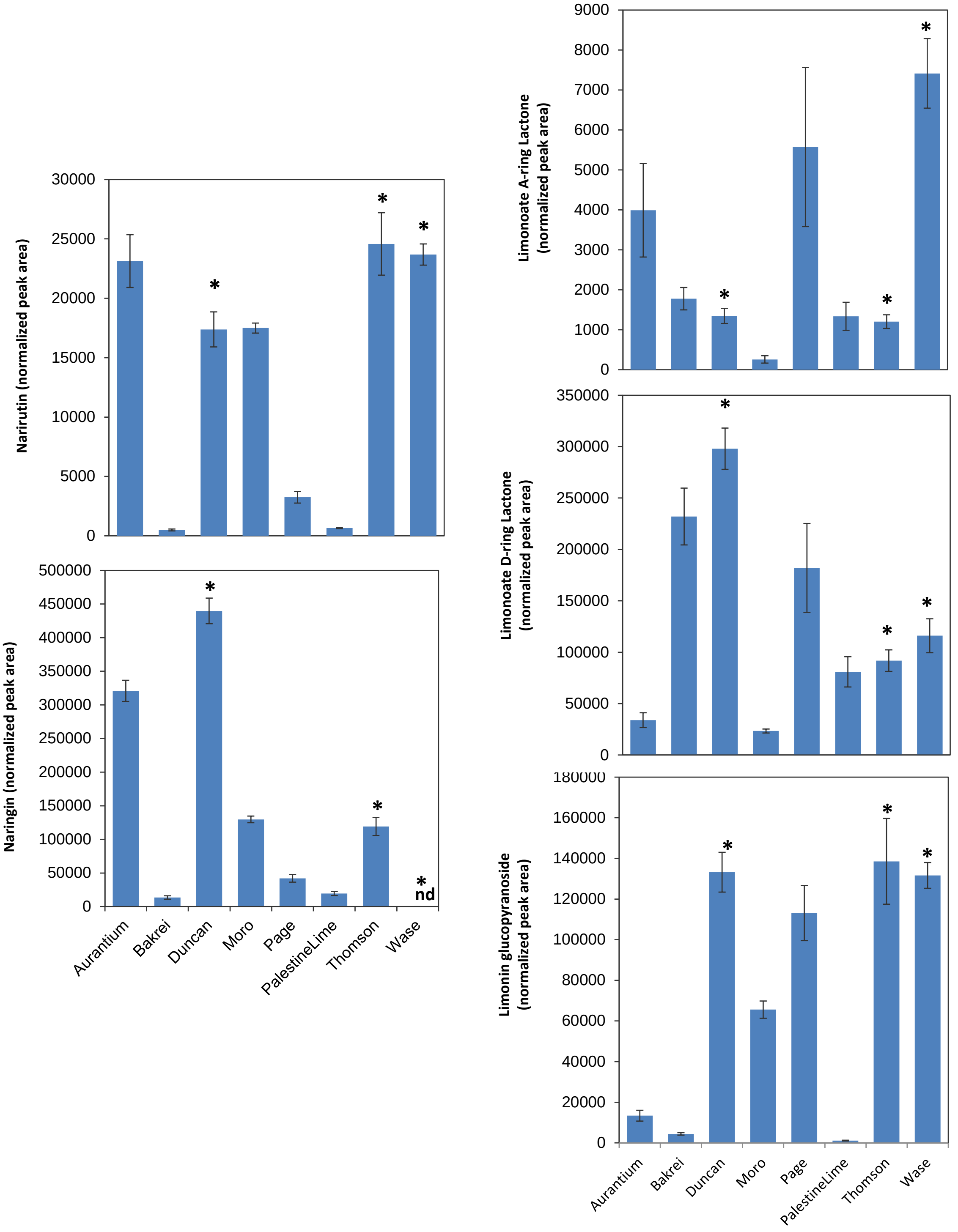

Supplementary Figure S4. Scores 2D scatter plot after PLS-DA analysis of specialized metabolite profiles from albedo and pulp of Duncan grapefruit (a), Wase mandarin (b) and Thomson navel orange 20, 80, 110 and 200 days after full bloom in positive electrospray ionization. The dashed line separates albedo and pulp samples.

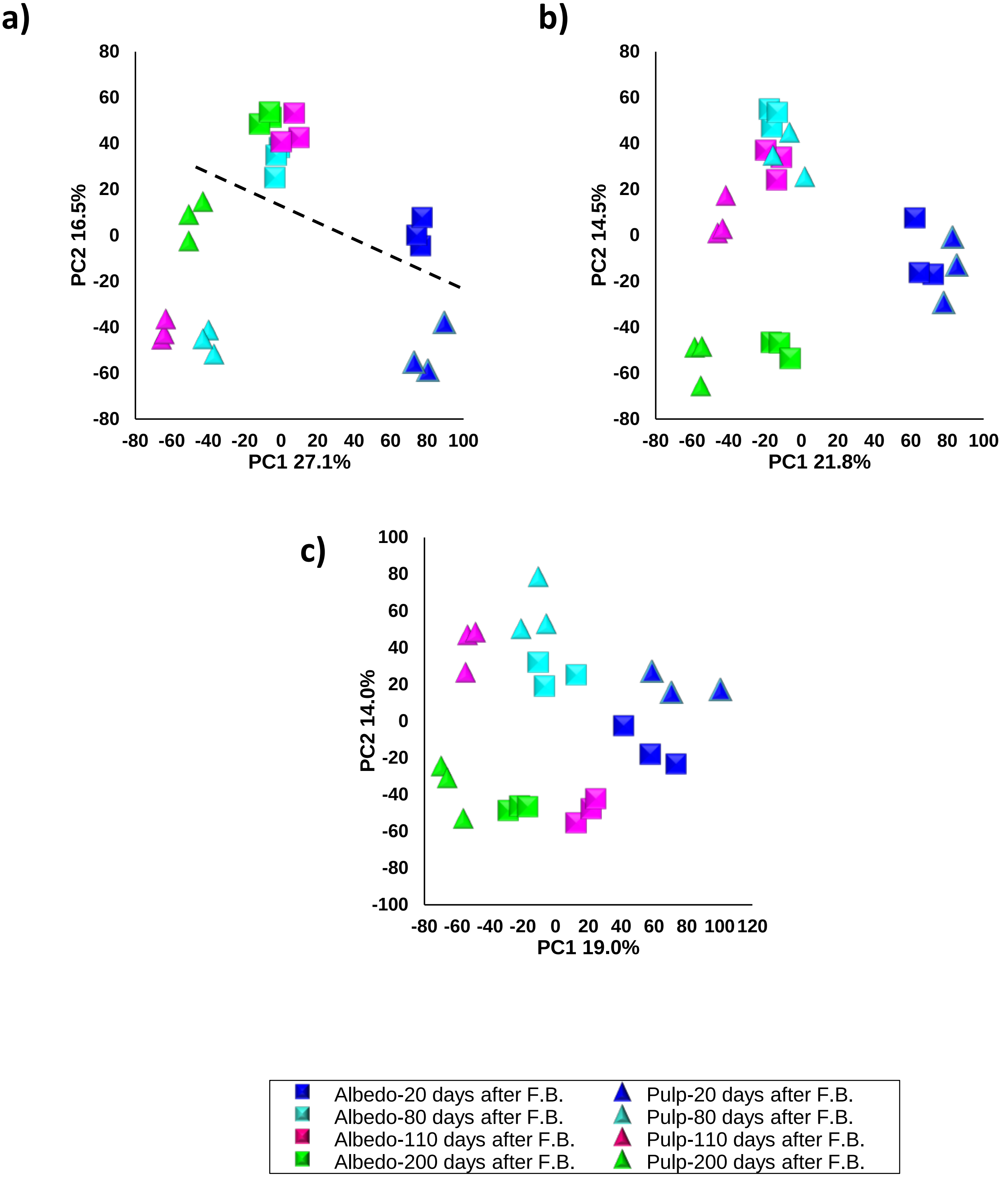

Supplementary Figure S5. Expression levels of genes involved in biosynthesis of flavonoids and limonoids. See Supplementary Material and Methods for details on total RNA extraction and qRT analyses.

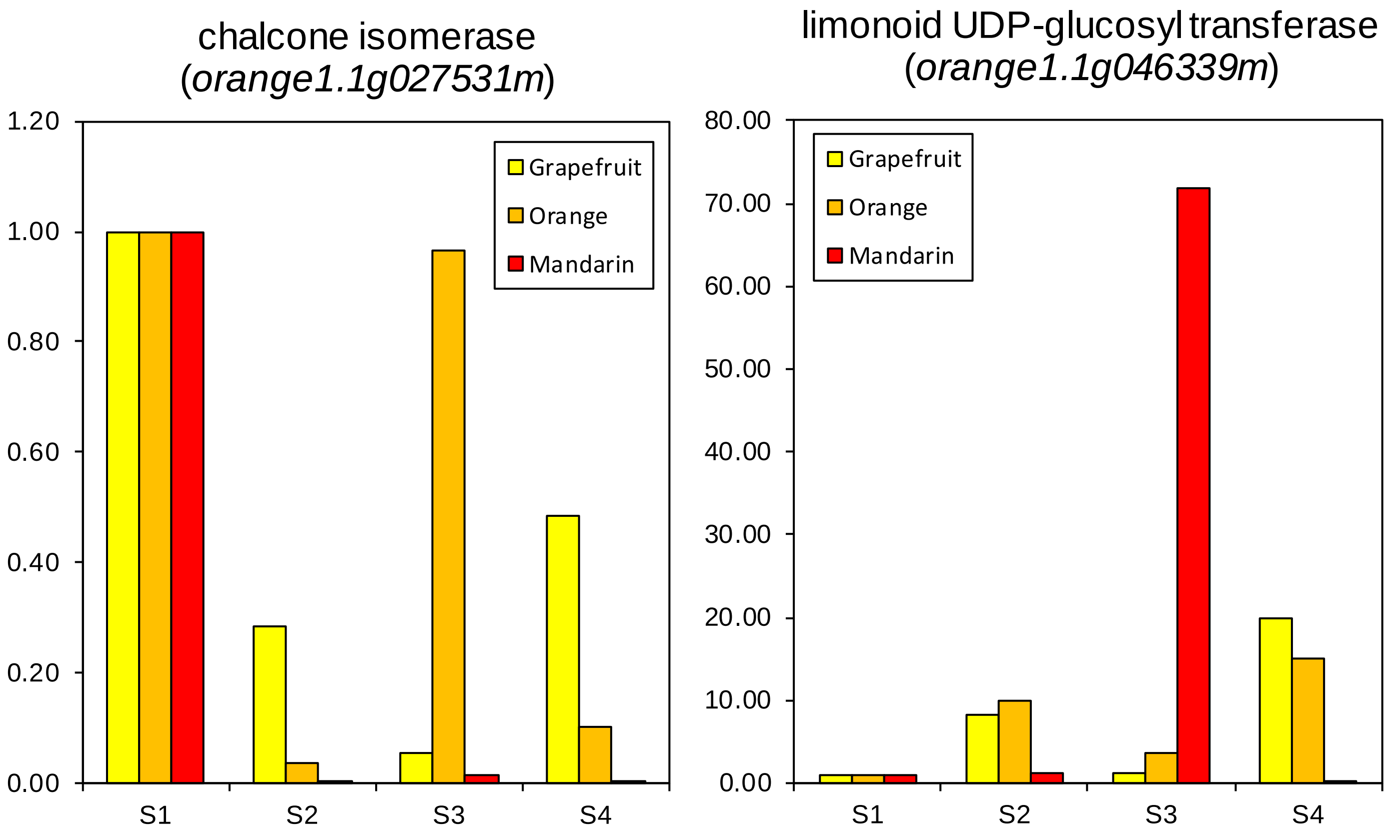

Supplement: Supplementary file 1 [file ijms-20-01245-s001.zip › Figures S1-S5.pdf]
