# Supplementary material for: Developmental Stage- and Genotype-Dependent Regulation of Specialized Metabolite Accumulation in Fruit Tissues of Different Citrus Varieties
Source: Int J Mol Sci. 2019 Mar 12;20(5):1245. doi: 10.3390/ijms20051245 (PMC6429498; doi:10.3390/ijms20051245)
Supplement: Supplementary file 1 [file ijms-20-01245-s001.zip › Supplementary Materials and Methods S1.pdf]

Supplementary Materials and Methods S1

Total RNA isolation, cDNA synthesis and qRT-PCR analyses

Total RNA was extracted from pulp tissues using the RNeasy minikit (Qiagen, Valencia, CA, USA) following manufacturer's intructions and subsequently resuspended in RNAase-free water. RNA purity and concentration were assessed by means of spectrophotometric analysis (Nanodrop, Thermo Scientific, Wilmington, DE, USA). Finally, single strand cDNA was synthesized from 1 μg total RNA using Primescript retrotranscriptase (TaKaRa Bio, Inc. Japan) and oligo(dT) primers.

Specific Forward (Fw) and Reverse (Rv) primers for qRT-PCR analyses were designed using primer3 plus (http://bioinfo.ut.ee/primer3-0.4.0/) software using orthologous sequences of the target genes retrieved from the *Citrus sinensis* genome (https://phytozome.jgi.doe.gov/pz/portal.html#!info?alias=Org_Csinensis), listed in the table below. In silico qRT-PCR evaluation of designed primers was carried out with IDT oligo analyzer (http://eu.idtdna.com/analyzer/applications/oligoanalyzer/) using the following parameters: Tm around 60 °C, amplicon length of 125–200 bp, primer length of 18–22 nucleotides with an optimum at 20 nucleotides and, finally, a GC content of 45–55%. Amplicon specificity was assessed by performing melting-curve analyses on each run.

qRT-PCR analyses were carried out in a StepOne Real-Time PCR system (Applied biosystems Inc, Foster City, CA, USA). The reaction mixture contained 1 μl of cDNA, 5μl of SYBR Green (Applied Biosystems) and 1 μM of each gene-specific primer pair in a final volume of 10 μl. The following thermal profile was set for all amplifications: 95°C for 30 s followed by 40 cycles of 95°C for 5s and 60°C for 30 s. Three technical replicates were analyzed on each biological replicate. The expression of all genes was normalized against that of two endogenous control genes (Tubulin and Actin). Relative expression levels were calculated by using REST software (Pfaffl MW. 2002. Relative expression software tool (REST© for group-wise comparison and statistical analysis of relative expression results in real-time PCR. Nucleic Acids Res 30: 1–10).

List of primers used for qRT-PCR analyses.

**Gene Name**	**Citrus sinensis genome**	**Primers**
**Chalcone isomerase**	orange1.1g027531m	Fw GTTCACGGCGATAGGAGTGT
		Rv TTCGGCAACCTTCTCTGAGT
**Limonoid UDP-glucosyl transferase**	orange1.1g046339m	Fw CCTCGGAGAATCCAAATTCA
		Rv AGGACCGACAGGTCGTATT
