# Supplementary material for: Developmental Stage- and Genotype-Dependent Regulation of Specialized Metabolite Accumulation in Fruit Tissues of Different Citrus Varieties
Source: Int J Mol Sci. 2019 Mar 12;20(5):1245. doi: 10.3390/ijms20051245 (PMC6429498; doi:10.3390/ijms20051245)
Supplement: Supplementary file 1 [file ijms-20-01245-s001.zip › Tables S1 and S2.pdf]

Supplementary Table S1. List of genotypes included in this study

Cultivar name	Plant species	Fruit type	Harvesting period (Days after Full Bloom)	TSS (°Brix)	TA (%)
Wase	*Citrus unshiu* cv. Wase	Mandarin	20, 80, 110, 200	13.80	0.94
Thomson	*Citrus sinensis* cv. Thomson navel	Navel orange	20, 80, 110, 200	11.80	1.52
Duncan	*Citrus paradisi* cv. Duncan	Grapefruit	20, 80, 110, 200	11.10	2.78
Moro	*Citrus sinensis* cv. Moro	Blood orange	20, 80, 110, 200	11.20	1.45
Aurantium	*Citrus aurantium* L.	Sour orange	20, 80, 110, 200	9.70	5.60
Palestine	*Citrus limettioides* cv. Palestine	Lime	20, 80, 110, 200	9.80	0.11
Bakraei	*Citrus reticulata* Blanco × *Citrus limettioides* Tan.	Mandarin	20, 80, 110, 200	7.80	0.07
Page	(*Citrus paradisi* cv. Duncan × *Citrus reticulata* cv. Dancy) × *Citrus clementina*	Mandarin	20, 80, 110, 200	12.80	1.47

Supplementary Table S2. Identification of metabolites as extracted from PLS-DA. VIP: Variable Importance; ID level: a) co-injection with standard, b) mass spectrometry data matches those in databases (metlin or HMDB), c) tentatively annotated based on mass spectrometry data and database search (See Material and Methods section for details). (*) p-value after one-way ANOVA using genotype as factor.

	**Positive electrospray (ESI+)**					**Negative electrospray (ESI-)**						
**Annotation**	**mz precursor**	**MS/MS**	**Rt [min]**	**Fragment annotation**	**VIP**	**mz**	**MS/MS**	**Rt [min]**	**Fragment annotation**	**VIP**	**ID level**	***p-value****
Naringenin	273.0700	153.0300	9.03	[M-C8H8O]+	-	271.0624	151.0055<br>119.0604	9.03	[M-C8H7O]-<br>[M-C7H3O4]-	1.33	a	<0.001
Myricetin	319.054	153.041	7.30	[M-C4H6O7]+	1.20	317.0342	179.99<br>151.0136<br>137.0183<br>109.0171	7.27	[M-C6H7O4]-<br>[M-C2H4]-<br>[M-O]-<br>[M-CHO2]-	1.44	a	<0.001
Nobiletin	403.1396	388.1245<br>358.0729<br>330.0801<br>301.0784<br>211.043<br>183.049	10.96	[M-CH3]+<br>[M-C2H6]+<br>[M-CO]+<br>-<br>-<br>-	1.34	nd					a	<0.001
Diosmin	609.1805	303.0976<br>177.0500<br>153.0200	7.72	[M-Hexose]+<br>[M-C6H3O3]+<br>[M-C9H10O2]+	1.03	607.1660		6.89	[M-H]-	0.95	a	<0.001
Isosinensetin	373.1289	343.1197	9.75	[M-C2H6]+	-	nd					c	<0.001
Sinensetin	373.1290	343.1199	10.39	[M-C2H6]+	1.16	nd					c	<0.001
Tangeretin	373.1291	343.1199	11.60	[M-C2H6]+	1.42	nd					c	<0.001

Eriodictyol rutinoside #1	451.1247	289.0738	5.35	[M-Hexose]-	1.15	595.1615		5.39	[M-H]-	1.01	c	<0.001
						287.0569		5.33	[M-rutinoside]+			
Eriodictyol rutinoside #2	597.1788	289.0732	6.14	[M-rutinoside]+	0.97	595.1662	287.0574	6.17	[M-rutinoside]-	0.97	c	<0.001
Isorhamnetin-3-O-rutinoside	625.1758	479.1195	6.76	[M-Deoxyhexosie]+	1.50	623.1614		6.73	[M-H]-	1.36	b	<0.001
		317.0669		[M-Hexose]+	1.46	315.0519		6.74	[M-rutinoside]-	1.23		
Isorhamnetin rutinoside hexoside	787.2267	317.0568	5.40	[M-rutinoside]+	1.14	785.2158		5.36	[M-H]-	1.25	b	<0.001
		479.1185		[M-hexoside]+	1.29							
Isorhamnetin rutinoside deoxyhexoside	771.2325	317.0663	6.02	[M-rutinoside-deoxyhexoside]+	1.40	769.2181		6.06	[M-H]-	1.33	b	<0.001
Kaempferol diDeoxyhexoside hexoside	741.2224	287.0559	5.98	[M-Deoxyhexoside-Hexoside]+	1.47	739.2099		6.07	[M-H]-	1.32	b	<0.001
		595.1649		[M-Deoxyhexoside]+								
Kaempferol Deoxyhexoside hexoside	595.1647	287.0565	6.63	[M-Deoxyhexoside-Hexoside]+	1.09	593.1558	285.0419	6.64	[M-Hexoside-Deoxyhexoside]-	1.30	b	<0.001

		449.1100	6.64	[M-deoxyhexoside]+	1.20							
Kaempferol Caffeoyl Hexoside Deoxyhexoside	757.2167	611.1477	5.34	[M-Deoxyhexose]+	1.38	755.2044	641.1742	5.33	-	1.31	b	<0.001
		449.1133		[M-Caffeoyl]+								
		287.0644		[M-Hexose]+								
Kaempferol hidoxymethyl glutaryl (HMG)-glucoside tentative	287.0565		7.13	Kaempferol moiety	1.15	591.1360	161.0583	7.08	hydroxymethyl glutarate moiety	0.92	c	<0.001
Quercetin hexoside rutinoside	773.2128	465.1050	4.99	[M-Rutinoside]+	1.31	771.2002		4.99	[M-H]-	1.28	b	<0.001
		303.0500		[M-Hexose]+								
Poncirin	595.2021	577.1910	7.91	[M-H2O]+	1.12	593.1874	285.0776	7.91	[M-rutinoside]-	0.97	b	<0.001
		449.1449		[M-Deoxyhexose]+								
		433.1497		[M-Hexose]+								
		287.0922		[M-Hexose]+								
Narirutin	581.1857	435.1293	5.51	[M-Deoxyhexose]+	1.12	579.1449		5.77	[M-H]-	1.31	a, b	<0.001
		273.0766		[M-Hexose]+								
Naringin	581.1864		6.64	[M+H]+	1.07	579.1711		6.65	[M-H]-	0.93	a	<0.001

	435.1291		6.63	[M-Deoxyhexose]+	1.15							
	273.0768		6.67	[M-Hexose]+	1.00							
Deacetyl Nomilinic acid glycoside tentative	693.2733	491.2279	6.50	[M-Hexose]+	0.98	669.2754		6.53	[M-H]-	0.96	c	<0.001
Deacetyl Nomilin glycoside	675.2619	473.2176	6.96	[M-Hexose]+	1.01	651.2673		6.96	[M-H]-	0.96	c	<0.001
Nomilin	515.2286	487.2336	11.14	[M-CO]+	0.94	513.2118		11.14	[M-H]-	1.03	b	<0.001
Nomilinic acid glycoside	712.3161	455.2066	7.39	[M-CH3COOH]+	1.23	711.2874		7.36	[M-H]-	1.02	b	<0.001
		515.2279		[M-CH3]+								
		533.2380		[M-Hexose]+								
Nomilin A-ring lactone	533.2381		10.53		1.04	531.2236	515.1915	10.53	[M-CH3]-	1.03	b	<0.001
							489.2155		[M-CH3CO]-	1.11		
							469.1865		[M-CH2O2]-	1.07		
							427.2129		[M-CH2O3]-	1.16		
							411.1453		[M-CH2O4]-	1.03		
Nomilin glycoside tentative	735.2791	533.2380	7.35	[M-Hexose]	1.07	711.2874		7.36	[M-H]-	1.02	c	<0.001
		515.2279	7.37	[M-Hexose]-CO]+	1.15							
		488.2377	7.39	-	1.21							
		469.2226	7.37	[M-CH2O2]+	1.19							
		457.2096	7.39	-	1.18							

		455.2066	7.40	[M-C2H4O2]+	1.09							
		427.2121	7.39	[M-CO]+	1.23							
		419.1991	7.39	-	1.18							
Obacunone	455.2076	-	11.14	[M+H]+	0.91	nd					c	<0.001
Obacunoic acid	473.2170	429.2274	10.26	[M-CO]+	1.50	nd					b	<0.001
Obacunone glycoside	455.208		7.71	[M-Hexose]+	1.12	633.2555	499.1799	7.73	[M-Hexose+HCOOH]-	1.41	c	<0.001
							517.2292		[M-Hexose fragment]-			
Ichangin	489.2120		9.65	[M+H]+	1.38	487.2120	427.1880	9.61	[M-C2H4O2]-	1.21	c	<0.001
Limonoate A-ring lactone tentative	489.2120		9.81		-	487.2120	427.1880	9.81	[M-C2H4O2]-	-	c	<0.001
							383.1847		-			
Limonin	471.2018	381.2074	10.56	[M+H-CO2]+	1.24	469.1935		10.53		-	a	<0.001
		383.1955		[M+H-COCH2]+								
		397.2022		[M+H-CO]+								
		425.1964		[M+H-HCOOH]+								
		443.2070		[M+H-CO]+								
		453.1923		[M+H-H20]+								
Limonin 17 beta D glucopyranoside	674.2515	425.1964	6.38	[M+H-HCOOH]+	1.10	649.2501	605.2612	6.38	[M-CO2]-	1.03	a, b	<0.001
		443.2072		[M+H-CO]+	1.19							
		453.1916		[M+H-H20]+	1.10							
		471.2014		[M-Hexose]	1.10							
Tryptophan	205.0988	144.0837	4.18	[M+H-CO2]+	1.13	203.0840	142.0670	4.16	[M-NH3]-	1.12	b	<0.001

		146.0618		[M+H-COCH2]+			116.0523	4.17	-			
		170.0605		[M+H-H20]+			159.0943	4.16	[M-CO2]-			
		188.0718		[M-NH3]+								
Phenylalanine	166.0869	103.0539	2.30	[M-NH3]+	1.34	164.0712		2.33		0.93	b	<0.001
				[M-HCO2]+								
Abscisic acid	247.1348	201.1286	8.18	[M-HCOOH]+	1.07	263.1303		8.35		0.95	a	<0.001
		205.1240		[M-COCH2]+								
		219.1395		[M-CO]+								
		229.1249		[M-H20]+								
Abscisic acid glycosyl ester	427.1023	201.1285	6.60	[M-HCOOH]+	1.46	425.1821	285.0419	6.66	[M-Hexose+Na]-	1.07	a	<0.001
		205.1228		[M-COCH2]+								
		219.1416		[M-CO]+								
		229.1251		[M-H20]+								
		247.1345		[M-Hexose-H2O]+								
12-oxophytodienoic acid	293.2105	275.2005	13.05	[M-H2O]+	1.12	291.2331	247.1989	13.05	[M-HCO2]	0.90	a	<0.001
							165.1235					
Dihydroconiferyl alcohol	183.1031	147.0462	6.35	[M-2xH2O]+	1.42	181.0878	119.0517	6.35	[M-C2H6O2]]-	1.32		<0.001
		165.0560		[M-H2O]+			137.0978		[M-CO2]-			
							163.0420		[M-H2O]-			
caffeic acid	181.0599	163.0495	5.40	[M-H2O]+	1.16	179.0379	135.0490	5.47	[M-CO2]-	1.35	a	<0.001
sinapinic acid	225.0763	119.0526	6.65	[M-C2H2]+	1.26	223.0626	208.047	6.71	[M-CH3]-	1.22	a	<0.001
		145.0329		[M-CH2O]+			179.066		[M-CO2]-			

		175.0412		[M-CH4O]+			164.051		[M-CH3]-			
		207.0668		[M-H2O]+								
chlorogenic acid	355.1035	163.0417	5.08	[M-C7H12O6]+	1.03	353.0883	191.0213	4.96	[M-Hexose]-	1.32	a	<0.001
Glucosyl salicylate	nd					299.0776	137.0264	3.88	[M-Hexose]+	0.98	a, b	<0.001
